# Exploring phages through play: creative 3D models for science engagement

**DOI:** 10.1099/acmi.0.001158.v3

**Published:** 2026-07-03

**Authors:** Maisie R. Czernuszka, George Dodgson, Andrew Martin, Michael Walsh, Ian B. Goodhead, Joanne L. Fothergill, Heather E. Allison, Chloë E. James

**Affiliations:** 1 School of Science, Engineering and Environment, University of Salford, SEE Building, University Road, Salford, M5 4WT, UK; 2 Department of Chemistry, Faculty of Science and Engineering, University of Manchester, Manchester Institute of Biotechnology, Princess Street, Manchester, M1 7DN, UK; 3 Institute of Infection, Veterinary and Ecological Sciences, University of Liverpool, Brownlow Hill, L69 7ZB, UK

**Keywords:** 3D printing, active learning, bacteriophages, creative science communication, education, public engagement

## Abstract

Phages are viruses that infect bacteria and have therapeutic potential due to their ability to selectively kill bacterial pathogens. Despite growing scientific and policy interest in phage therapy, public and professional understanding of phages remains limited, posing a barrier to wider clinical adoption. Here, we present the development and evaluation of open-source, 3D-printed microbial models designed to communicate core concepts in phage biology, including phage diversity, host specificity and life cycle differences between virulent and temperate phages. These tactile, compact models were tested across multiple public engagement events with diverse audiences. Survey data showed high usability and educational value, 90% of participants reported improved understanding of phage-bacteria interactions, and many expressed interest in learning more. Thematic analysis of qualitative feedback highlighted sustained engagement and prompted iterative model refinements to improve clarity and accessibility. These models offer a low-cost, scalable tool to support outreach and education around phage biology, including its applications in treating drug-resistant infections. By bridging the gap between complex scientific concepts and public understanding, they contribute to broader efforts to build awareness of phage-based alternatives to traditional antibiotics.

## Data Summary

The 3D model files are publicly available via MakerWorld [1,2], with direct access links provided in the Supplementary Materials. Survey questions and thematic summaries of qualitative feedback are also provided in the Supplementary Materials. Quantitative survey data are presented in Fig. 3.

## Introduction

Bacteriophages (phages) are viruses that infect bacteria and are the most abundant biological entities on Earth, with an estimated 10³¹ particles globally [3]. These viruses are extremely diverse and exhibit different life cycles, which influence their roles in both microbial ecology and medicine [4]. Virulent phages follow a lytic cycle, infecting and rapidly killing bacterial cells to release new phage particles [4]. Temperate phages, by contrast, can also enter a lysogenic cycle, integrating into the bacterial genome as prophages. While integrated, they can provide the host with adaptive traits, such as antibiotic resistance, and protection from further phage infections [4].

Phage therapy refers to the use of bacteriophages to treat bacterial infections. Given the differences in phage life cycles, virulent phages are generally favoured over temperate phages because they reliably lyse bacterial cells and reduce the risk of horizontal gene transfer [4,6]. Although practised in parts of Eastern Europe for decades, phage therapy is currently only used as a last-resort compassionate-use treatment in most other regions, largely due to poorly defined regulatory frameworks [7,8]. However, this therapeutic has gained increased interest as antimicrobial resistance (AMR) accelerates globally, with ~5 million deaths caused by antibiotic resistance in 2019 alone [9]. This momentum is reflected in several recent large-scale studies and clinical efforts [10,11]. Furthermore, in the UK, government-level attention has increased, including recent parliamentary inquiries into the antibacterial potential of phages, as well as the incorporation of phage therapy into the 5-year national action plan to combat AMR [12,13].

Yet despite this growing scientific and policy interest, public understanding of phages and their medical applications remains limited [8,13]. The recent UK parliamentary inquiry into phage therapy identified a widespread lack of awareness not only among the general public but also within the clinical community [13]. For phage therapy to be more widely adopted in healthcare systems, it is essential that clinicians and other healthcare professionals are equipped with accurate, accessible information [13,14]. Public perception presents another challenge; in the aftermath of the COVID-19 pandemic, the word ‘virus’ can evoke fear or mistrust [8,15]. McCammon *et al.* (2023) found that the strategic avoidance of terms like ‘kill’ and ‘virus’ increased public acceptance of phage therapy as a treatment preference [8]. Hence, these findings highlight the need to educate and reframe the role of phages in medicine in a way that is both scientifically accurate, transparent and publicly accessible. For this purpose, engaging and accessible outreach tools are urgently needed.

To address this gap, we have developed open-source 3D-printed microbial models and accompanying learning resources that illustrate how phages interact with their bacterial hosts. Specifically, we have separate designs to represent virulent and temperate phages, helping to communicate the distinction between phage types and their relevance to therapy. We have used these models across a range of public engagement events with diverse audiences, gathering feedback to refine both the designs and the way they are presented. These tools aim to support both conceptual understanding and critical discussion of the therapeutic roles of phages, helping to bridge the divide between scientific research and public awareness. Here, we present our approach, evaluate its impact on public understanding and reflect on how such tools can support science communication around AMR and emerging phage-based therapies.

## Methods

Ethical approval for this study was obtained from the University of Salford.

### Identifying interest

**Fig. 1.**
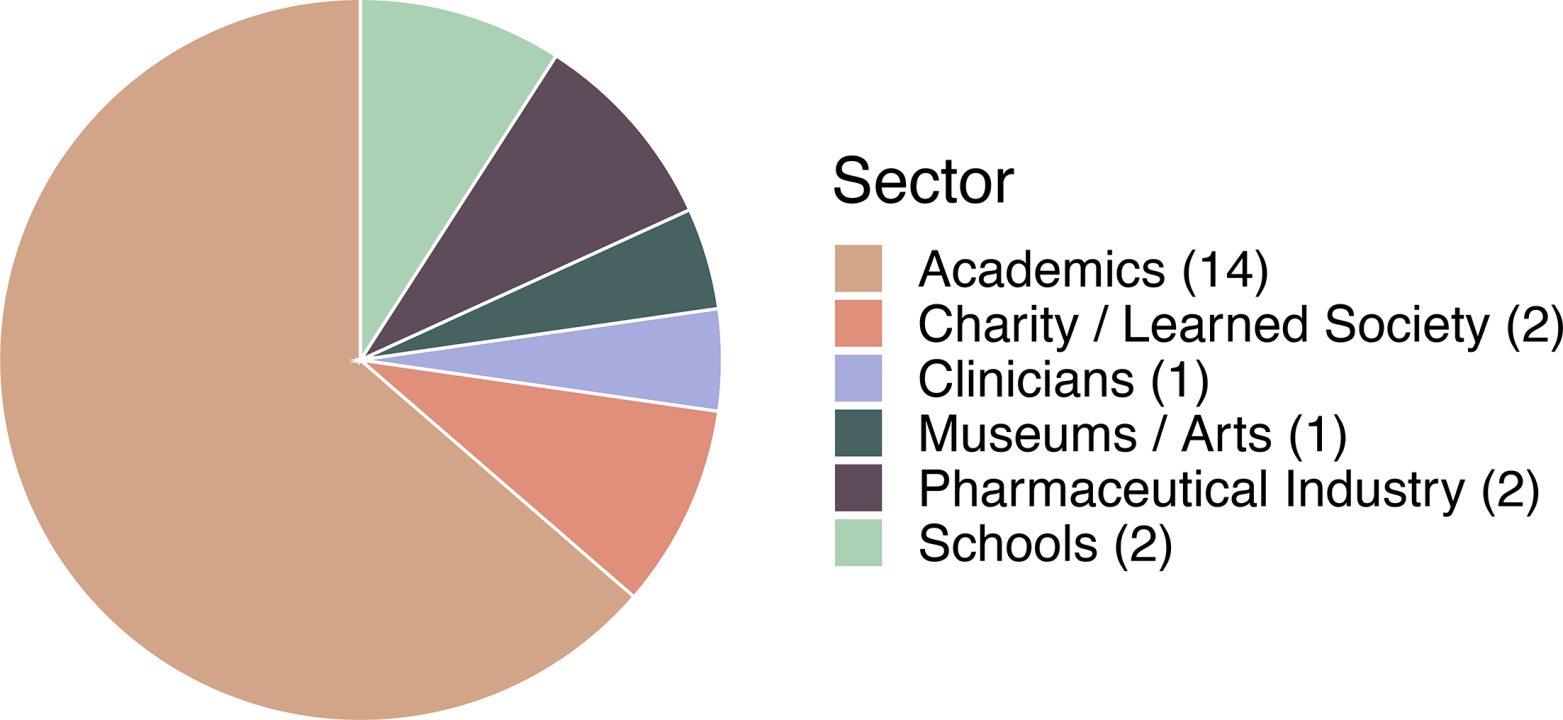
Stakeholder representation by sector. Stakeholders (*n*=22) were grouped by sector, including academia, industry, healthcare, education and outreach. Each segment represents an individual stakeholder identified during initial engagement activities. ‘School’ stakeholders correspond to individual secondary school educators.

We began by identifying interest from 22 stakeholders, including phage academics, industry researchers and secondary school educators, to assess the demand for accessible tools to communicate phage biology (Fig. 1). Stakeholders were identified through existing academic, industry and outreach networks, including interactions at scientific conferences and public engagement events. They were subsequently contacted by email to confirm participation. These early interactions aimed to gauge interest in the development and use of 3D-printed models as educational tools, to identify potential collaborators for testing and feedback and to inform the educational focus of the models. Collectively, these discussions indicated a clear demand for engaging and interactive resources to support public understanding of phage biology and therapeutics.

### Development of 3D-printed models

We developed two open-source 3D-printed microbial models, representing virulent and temperate phage infection, respectively. These designs are publicly available on MakerWorld and are cited here [1,2], with direct access links provided in the Supplementary Materials. These were inspired by larger, more complex prototypes previously designed by the *Microbial Puppet Masters* team and the Morson Maker Space, which were based on the mechanics of the board game TOMY *Pop-Up Pirate* [16,17]. While these earlier models were visually striking and mechanically effective, their size and complexity made them impractical for widespread use. To improve accessibility, we redesigned the models to be compact, intuitive and easily printable using standard 3D printers (e.g. Creality Ender 3 3D-printer with a current list price of £199. Each model was designed to be printed using a single filament type (e.g. ELEGOO PLA filament, 1.75 mm, 1 kg, with a current list price of £11.99; capable of printing four full units complete with accessories, requiring no multi-material setup or specialist equipment. Assembly was intentionally simplified with minimal tools and associated assembly instructions to encourage widespread adoption by the public and phage educators.

The models were designed to convey three core educational messages through their physical structure and functionality. These messages were selected to represent fundamental concepts in phage biology that are critical for understanding their therapeutic applications and were informed by early stakeholder discussions and common misconceptions observed during outreach activities:

- **Phage diversity:** represented by phage ‘keys’ with different tail fibre shapes. This is useful for priming conversations about the huge variety of phage types in existence that constitute a rich resource not only for novel therapeutic applications but also for other biotechnological advances [18]. With this concept, we could convey the wonder of scientific discovery and inspire young people to consider a career in science.
- **Host specificity:** shown by only one correctly shaped phage key successfully ‘infecting’ the model and triggering a response. This concept is crucial in demonstrating one of the key advantages of phage therapy over antibiotics, i.e. phages are highly specific, meaning they can target a particular pathogen without affecting the healthy microbiota [19]. This narrow specificity also represents a major challenge in matching the right phage to a particular bacterial target and the need to develop phage mixtures (cocktails) to effectively cover a sufficient range of common pathogenic strains of a given bacterial species [20]. This helps to explain the challenge of effective design of large clinical trials and thus why phage therapy isn’t already in general use worldwide [21].
- **Life cycle differences:** demonstrated by two contrasting responses: the virulent model ‘bursts’ open to simulate bacterial death by lysis and phage release, while the temperate model extends to represent lysogeny and the integration of phage DNA into the host genome [4]. This makes the bacterium appear ‘stronger’ by illustrating how lysogeny can enhance adaptation to the environment [4]. This concept again allows us to highlight the diverse nature of phages, how they have driven bacterial evolution and how understanding their biology has led to new branches of science [22]. The concept also demonstrates another key challenge for phage therapy developers, who must identify which phages are suitable as treatments and which could contribute to enhanced survival of the bacterial target [23].

All three concepts enhance dialogue with stakeholders about the future of phage research using high-throughput genomics, bespoke bioinformatics and AI tools to fast-track screening methods to identify the best phages for therapy.

### Public testing and outreach events

We tested the model prototypes at a range of public engagement events, reaching audiences across age groups (Table 1). Events included large-scale science festivals, museum exhibitions and targeted outreach sessions with school groups, providing opportunities to assess the models in varied learning environments.

**Table 1.** Summary of public outreach events and associated demographics using the microbial models

Event:	No. of attendees	Demographic	Reference
British Science Week at the Victoria Gallery and Museum	600	Young families	[32]
Local School Science Week	500	4–11 years old	–
University of Manchester Community Festival	~300	All ages	–
Breaking the Mould Summer School - University of Salford	30	17–18 years old	–
Royal Society Summer Science Exhibition on Tour at Jodrell Bank	1,300	All ages	[33]
Manchester Science Festival at the Science and Industry Museum	~20,000	Young families	–

To evaluate the models’ effectiveness, we collected both quantitative and qualitative feedback through surveys completed by members of the public and by event facilitators. Survey questions focused on usability (e.g. ease of use), educational value (e.g. whether participants felt they had learnt something new about phage biology) and interest in learning more about phages in the future (see Supplementary materials). Quantitative survey data were visualized in RStudio (v2023.09.1+494) using *ggplot2* [24]. Qualitative responses were thematically grouped into four key areas: improved understanding, sustained engagement, model usability and suggestions for improvement (see Table S1, available in the online Supplementary Material). These themes guided iterative improvements to the models, with representative feedback linked to corresponding design modifications (Table S1). Design changes included adding a stabilizing base to improve positioning and orientation, developing 3D visual assembly guides and a supporting tool to improve assembly and reassembly and modifying the receptor slot dimensions to increase the likelihood of successful ‘infections’. These refinements were informed directly by user experience and aimed at improving both educational impact and ease of use.

## Results and discussion

To assess the need for accessible phage education tools, we approached 22 stakeholders from a range of sectors to gauge interest in our proposed digital outreach package. These included representatives from academia, education, industry, healthcare and science communication. As shown in Fig. 1, academic researchers made up the largest group (*n*=14), highlighting a strong interest in interactive and engaging phage education tools within the research community.

**Fig. 2.**
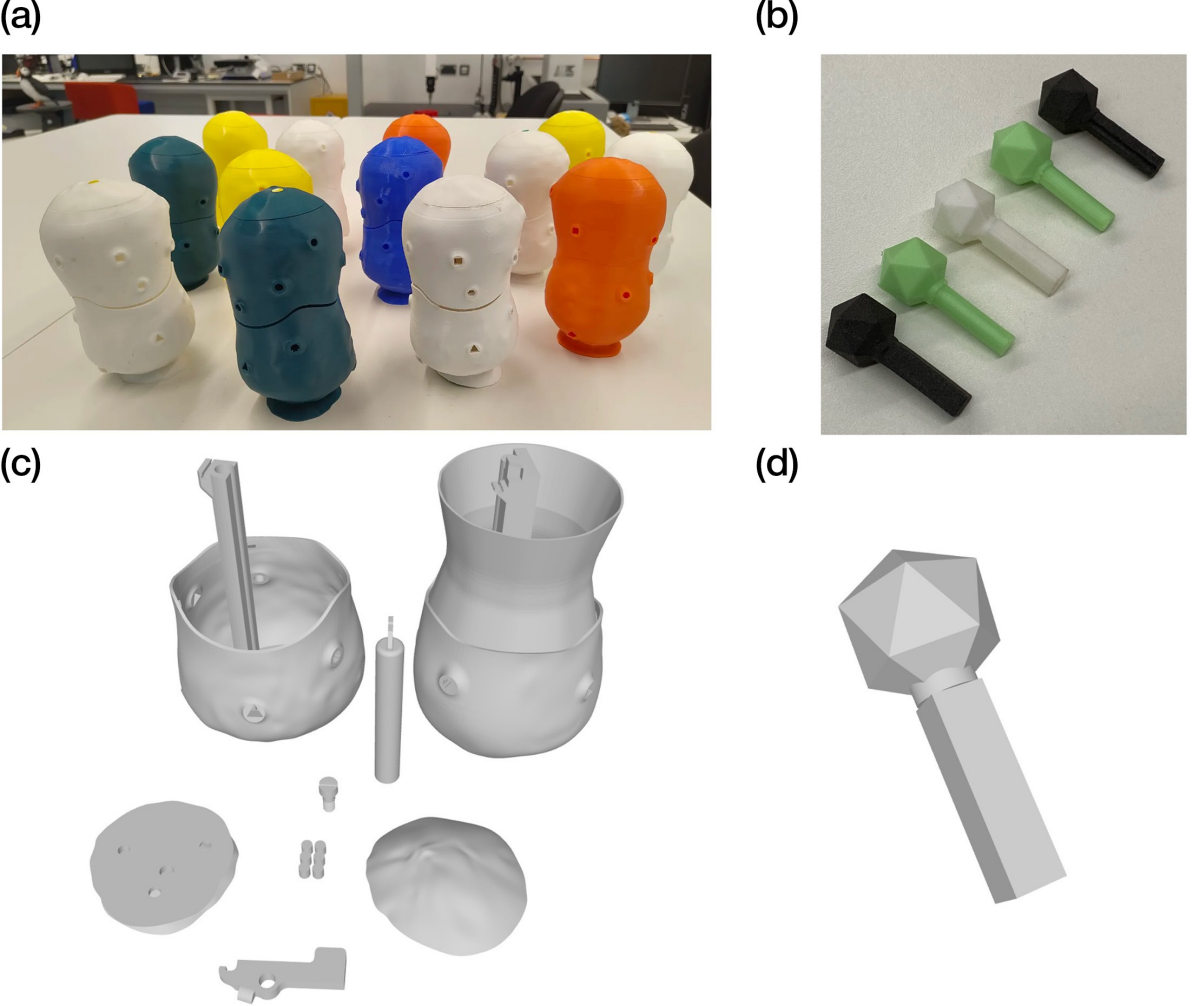
3D-printed bacterial models and associated phage ‘keys’. (**a**) Photos of printed bacterial models (both virulent and temperate) prior to infection and (b) printed phage key examples. (**c**) Individual printable components of lysogenic model and (d) phage key (visualized in online 3D Viewer) [34].

Building on this interest, we focused the design of our 3D-printed models around three key educational messages that are central to understanding phage biology and its therapeutic potential (Fig. 2). First, they showcase phage diversity through differently shaped ‘keys’, where the variation lies specifically in the tail fibre shapes, representing the vast array of phages in nature [25]. This diversity is one of phage therapy’s greatest strengths, offering a rich resource to target a wide range of bacterial hosts, including drug-resistant strains [26]. Secondly, the models demonstrate host specificity by illustrating how only a matching phage ‘key’, with the correct tail fibre shape, can successfully infect a bacterial host, emphasizing the need to carefully select phages for effective treatment [27]. Third, the models physically represent the different phage life cycles: virulent phages trigger a ‘burst’ simulating bacterial lysis and phage release, while temperate phages extend to represent lysogeny and integration into the bacterial genome [4]. This distinction clarifies why virulent phages are generally preferred for therapy, given their ability to actively kill bacteria, while temperate phages carry risks such as horizontal gene transfer [4]. Together, these features provide an engaging, hands-on tool suitable for audiences of all ages, including children, to explore and understand complex concepts in phage biology and its therapeutic applications.

**Fig. 3.**
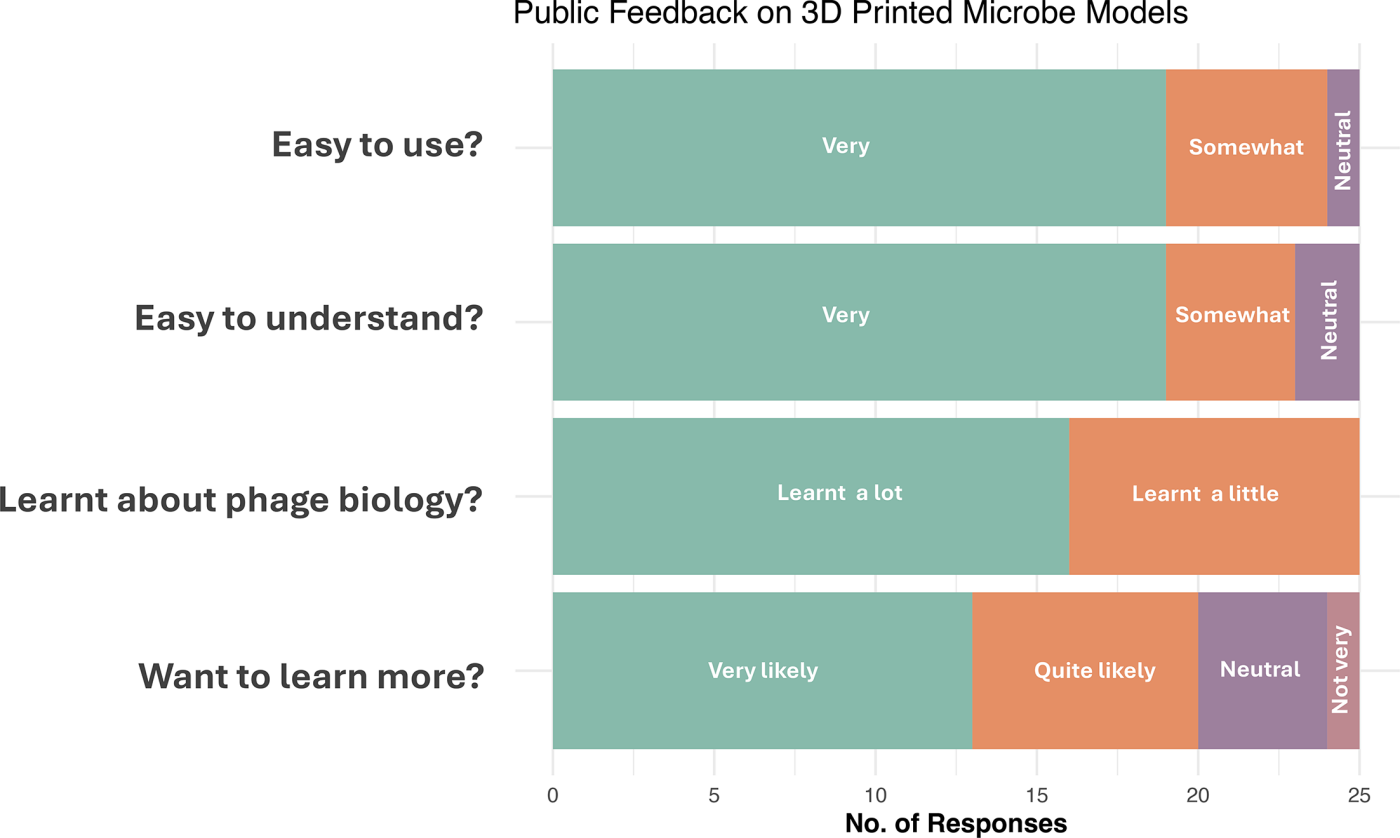
Participant feedback on 3D phage model usability and educational impact (*n*=25). See supplementary materials for question details.

These models were tested through their use at six large-scale public engagement events (Table 1), where participants interacted directly with them using phage ‘keys’. We used a survey to collect feedback from members of the public and volunteers on ease of use, clarity of message and potential impact, as shown in Fig. 3. Most respondents (96%) found the models easy to use, with 76% rating them as very easy and 20% somewhat easy. This ease of use is crucial for wider adoption in outreach settings, ensuring that facilitators and participants can engage without prior model knowledge. Similarly, 92% felt that the models improved their understanding of how phages interact with bacteria, with 76% finding this very clear. All participants reported learning something new about phages, including 64% who said they learnt a lot. Encouragingly, 81% indicated they were likely to explore microbiology further after engaging with the models, with over half (52%) very likely to do so. Together, these findings highlight the success of our model designs and support their use as engaging, accessible tools to communicate complex phage biology to diverse audiences.

In addition to structured survey responses, qualitative comments were collected across the public outreach events, offering insight into both the educational value and practical usability of the phage models. Thematic analysis of participant and volunteer comments revealed four key themes: improved understanding, sustained engagement, model usability and suggestions for improvement (see Table S1).

Improved public understanding and engagement emerged as a consistent theme, with many participants reporting newfound awareness and interest in phage biology. For example, one participant noted, ‘*I’ve never found biology so interesting before*’. Notably, visitors with limited prior knowledge also expressed surprise at how engaging the topic was, with comments such as ‘*Really interesting. As an adult, it was a concept even new to me*’. Sustained engagement was also evident, with some participants returning multiple times to explore further or attend related talks, as reflected in comments such as ‘*Came back again the next day to attend our public lecture*’. These findings align with the intended goal of the models as tools to shed light on phage biology and promote broader microbiology literacy. Model usability was another prominent theme. Overall, the models were well-received for their ease of use and accessibility across age groups. Feedback indicated that the smaller redesigned versions were preferred over previous larger models, especially among children who found them easier to manipulate. However, some users experienced difficulties, such as incorrectly holding the models or trouble aligning the phage ‘keys’ with the receptor slots, which occasionally limited the intended interactive effect. These observations underline the importance of clear design cues for educational tools used in diverse settings. These usability challenges directly informed targeted design modifications, including improvements to model positioning and orientation, assembly guidance and interaction success (Table S1).

The final theme was suggestions for improvement. Participants and facilitators provided practical recommendations to enhance the models’ robustness and user experience. Common suggestions included adding stabilizing bases to prevent tipping, improving internal mechanisms to reduce jamming and incorporating clearer visual guides to assist correct handling. Additionally, simplifying the assembly and reset process was highlighted as important for maintaining usability during busy public events. Many of these points informed targeted iterative improvements to the models, with representative feedback linked to corresponding design changes (Table S1).

Together, these thematic insights confirm that the 3D-printed phage models serve as effective, engaging educational tools while also providing valuable guidance for future refinements to maximize their impact in outreach and learning environments.

These models form one part of a broader suite of public engagement activities developed by our *Microbial Puppet Masters* team to communicate phage biology and therapeutics to diverse audiences [16]. Alongside virtual reality experiences, craft-based workshops and larger 3D installations, these new open-source prototypes offer a distinctively accessible and interactive format – compact, tactile and easily reproducible – that anyone with access to a 3D printer can use in classrooms, community events, science festivals and professional settings. By providing a low-cost, hands-on tool for demonstrating phage biology, these models can be produced for ~£3–5 per unit, depending on material and printing settings, and are well suited for education across age groups, as well as training and engagement with healthcare professionals, policymakers and other stakeholders. Indeed, we have used the models to support dialogue on phage therapy with local government, as well as politicians in the House of Lords.

While the primary goal was to introduce key concepts in phage biology, such as phage diversity and specificity, participants also took away messages we had not explicitly planned but which proved valuable. For example, the act of assembling the phage and ‘infecting’ a bacterial cell with it often led participants to ask whether phages multiply once inside, prompting discussions about phage replication. Others focused on the challenge of ‘matching’ phages to the right bacterial target, highlighting a central difficulty in personalized phage therapy [27,28]. These unprompted moments underscore the value of 3D models in science communication; by doing and manipulating, participants generated their own questions and interpretations. This aligns with broader pedagogical principles of active learning which has repeatedly been shown to deepen understanding and support long-term retention [29,31].

While the models offer an accessible and engaging way to communicate phage biology, their strength lies in conveying fundamental concepts, such as phage diversity, specificity and life cycle differences, through physical interactions. Rather than replicating the full complexity of phage-host dynamics, the models provide a foundation that can unlock more nuanced dialogue and deeper understanding. However, we have not yet evaluated whether users require prior phage knowledge to effectively use and deliver education with these models, which may impact their accessibility for non-expert educators or audiences. Future work should explore how these models can be integrated more systematically into outreach and education, potentially co-developed with teachers or community groups to align with curriculum needs or local interests. Beyond phage biology, the simple open-source design could be adapted to illustrate other microbiological processes, opening wider opportunities for creative, low-cost learning tools. As part of the next phase, we plan to distribute learning packages, comprising the models alongside associated educational resources, to stakeholders across our network, including leading figures in the phage community. Feedback from these users will help refine both the models and accompanying materials, ensuring that they meet the needs of diverse audiences and maximize their utility in phage education and advocacy.

Such tools are particularly relevant for improving societal understanding of alternative antimicrobial strategies at a time of growing concern about antibiotic resistance [9]. While public awareness of antibiotics is relatively high, phage therapy remains largely unfamiliar. Models like these offer a route to destigmatize the science, helping audiences grasp not just what phages are but also how they might, and might not, work as treatments. As such, they may contribute to more informed public dialogue about the possibilities and limitations of phage-based medicine.

## Supplementary material

10.1099/acmi.0.001158.v3 Table S1.

## Abbreviations

AMR: antimicrobial resistance

## References

[1] Salford MS (2025). Phage lysogenic [3D Model]. https://makerworld.com/en/models/1413039-phage-lysogenic#profileId-1467109.

[2] Salford MS (2025). Phage lytic [3D Model]. https://makerworld.com/en/models/1418843-phage-lytic#profileId-1473771.

[3] Keen EC (2015). A century of phage research: bacteriophages and the shaping of modern biology. Bioessays.

[4] Ranveer SA, Dasriya V, Ahmad MF, Dhillon HS, Samtiya M (2024). Positive and negative aspects of bacteriophages and their immense role in the food chain. NPJ Sci Food.

[5] Abedon ST, García P, Mullany P, Aminov R (2017). Editorial: phage therapy: past, present and future. Front Microbiol.

[6] Weber-Dąbrowska B, Jończyk-Matysiak E, Żaczek M, Łobocka M, Łusiak-Szelachowska M (2016). Bacteriophage procurement for therapeutic purposes. Front Microbiol.

[7] Yang Q, Le S, Zhu T, Wu N (2023). Regulations of phage therapy across the world. Front Microbiol.

[8] McCammon S, Makarovs K, Banducci S, Gold V (2023). Phage therapy and the public: Increasing awareness essential to widespread use. PLOS One.

[9] Murray CJL, Ikuta KS, Sharara F, Swetschinski L, Robles Aguilar G (2022). Global burden of bacterial antimicrobial resistance in 2019: a systematic analysis. The Lancet.

[10] Pirnay J-P, Djebara S, Steurs G, Griselain J, Cochez C (2024). Personalized bacteriophage therapy outcomes for 100 consecutive cases: a multicentre, multinational, retrospective observational study. Nat Microbiol.

[11] Uyttebroek S, Chen B, Onsea J, Ruythooren F, Debaveye Y (2022). Safety and efficacy of phage therapy in difficult-to-treat infections: a systematic review. Lancet Infect Dis.

[12] Department of Health and Social Care (2019). The UK’s Five-Year National Action Plan.

[13] House of Commons Science IaTC (2023). The antimicrobial potential of bacteriophages.

[14] Jones JD, Stacey HJ, Brailey A, Suleman M, Langley RJ (2023). Managing patient and clinician expectations of phage therapy in the United Kingdom. Antibiotics (Basel).

[15] Mahmood QK, Jalil A, Farooq M, Akbar MS, Fischer F (2023). Development and validation of the post-pandemic fear of viral disease scale and its relationship with general anxiety disorder: a cross-sectional survey from Pakistan. BMC Public Health.

[16] University of Salford Manchester (2025). Pulling back the curtain on Microbial Puppet Masters. https://scicomm.space/rs22.

[17] University of Salford Manchester (2025). Maker Space University of Salford Manchester. https://makerspace.salford.ac.uk.

[18] Jo SJ, Kwon J, Kim SG, Lee SJ (2023). The biotechnological application of bacteriophages: what to do and where to go in the middle of the post-antibiotic era. Microorganisms.

[19] Ross A, Ward S, Hyman P (2016). More is better: selecting for broad host range bacteriophages. Front Microbiol.

[20] Teklemariam AD, Al Hindi R, Qadri I, Alharbi MG, Hashem AM (2024). Phage cocktails - an emerging approach for the control of bacterial infection with major emphasis on foodborne pathogens. Biotechnol Genet Eng Rev.

[21] Moon K, Coxon C, Årdal C, Botgros R, Djebara S (2025). Considerations and perspectives on phage therapy from the transatlantic taskforce on antimicrobial resistance. Nat Commun.

[22] Stern A, Sorek R (2011). The phage-host arms race: shaping the evolution of microbes. Bioessays.

[23] Cook BWM, Hynes AP (2025). Re-evaluating what makes a phage unsuitable for therapy. *NPJ Antimicrob Resist*.

[24] Wickham H ggplot2.

[25] Dion MB, Oechslin F, Moineau S (2020). Phage diversity, genomics and phylogeny. Nat Rev Microbiol.

[26] Cui L, Kiga K, Kondabagil K, Węgrzyn A (2024). Current and future directions in bacteriophage research for developing therapeutic innovations. Sci Rep.

[27] Gaborieau B, Vaysset H, Tesson F, Charachon I, Dib N (2024). Prediction of strain level phage-host interactions across the *Escherichia* genus using only genomic information. Nat Microbiol.

[28] Venkataraman S, Shahgolzari M, Yavari A, Hefferon K (2025). Bacteriophages as targeted therapeutic vehicles: challenges and opportunities. Bioengineering (Basel).

[29] Freeman S, Eddy SL, McDonough M, Smith MK, Okoroafor N (2014). Active learning increases student performance in science, engineering, and mathematics. Proc Natl Acad Sci USA.

[30] Ginsburg M (2010). Improving educational quality through active-learning pedagogies: a comparison of five case studies. Educational Res.

[31] Allsop J, Young S, Nelson E, Piatt J, Knapp D (2020). Examining the benefits associated with implementing an active learning classroom among undergraduate students. J Learn Teach High Educ.

[32] Czernuszka M (2024). Unlocking the Secrets of Bacteriophages: A British Science Week Extravaganza. https://scicomm.space/diary/2024/5/21/unlocking-the-secrets-of-bacteriophages-a-british-science-week-extravaganza.

[33] Alsaadi SE (2024). Engaging with bacteriophages and celebrating diversity. https://microbiologysociety.org/blog/engaging-with-bacteriophages-and-celebrating-diversity.html?.

[34] Kovacs V (2025). https://github.com/kovacsv/Online3DViewer.

