# Supplementary material for: Exploring phages through play: creative 3D models for science engagement
Source: Access Microbiol. 2026 Jul 3;8(7):001158.v3. doi: 10.1099/acmi.0.001158.v3 (PMC13331358; doi:10.1099/acmi.0.001158.v3)
Supplement: Table S1. [file acmi-8-01158-s001.pdf]

Supplementary Materials:

Supplementary Materials - 3D Model Access

- Virulent model (lytic infection):
  - https://makerworld.com/en/models/1418843-bacteria-virulent-phage-infection-lytic#profileId-1473771
- Temperate model (lysogenic infection):
  - https://makerworld.com/en/models/1413039-bacteria-temperate-phage-infection-lysogenic#profileId-1467109

Supplementary Material - Public 3D Microbe Models Survey Questions:

1) How did you feel the models functioned during your interaction?
2) Did you models help you understand the "lock and key" interaction between bacteria and phages?
3) Did the models help you learn about bacteria and phages?
4) Do you expect to investigate microbiology more in the future after having used these models?
5) OPTIONAL: Any other thoughts or feedback on the 3D interactive models?

Supplementary Table 1. Thematic analysis of qualitative feedback

Theme	Representative Quotes	Source	Design Implication
**Improved understanding**	"Really interesting. As an adult, it was a concept even new to me!"	Public participant	-
	"I've never found biology so interesting before."	Teenage visitor	-
	"Found the talk really amazing – begged grandma to come back and learn more afterward."	Child (via parent)	-
	"I found that much more interesting than I thought I would."	Older adult visitor	-
	"Wow – this is amazing!"	Parent of young children	-
	"Excellent exhibit clearly communicating the science. Really enjoyable, thanks"	Public participant	-

**Sustained engagement**	"Came back again the next day to attend our public lecture."	Adult visitor	-
	"I'm noticing we are forming personal bonds with the audience – a sign of genuine interest."	Delivery team member	-
**Model usability**	"Most people, and the children especially, found the smaller models much easier to work with than the bigger ones."	Delivery team member	Adopted smaller, simplified models.
	"Children loved the new smaller models… would try and pop them open loads of times."	Parent (via team report)	Reinforced use of compact interactive designs.
	"People often held the model the wrong way up… which limited the effect of the phage bursting."	Delivery team observation	Added stabilising base and orientation cues.
	"More difficult to pop the cells open if the triangle shape was the correct key – was more of a problem for young children."	Delivery team observation	Adjusted receptor slots for easier interaction.
**Suggestions for improvement**	"When the models would accidentally de-assemble, the staff could not easily work out how to put it back together."	Delivery team observation	Added 3D visual assembly guides and tool.
	"One of the virulent models broke – the trigger fell out and we couldn't reassemble it."	Delivery team observation	Added assembly tool for easier reassembly.
	"Volunteers suggested designing a base to hold the smaller models as they would often fall over."	Volunteer feedback	Added stabilising base.
	"When refilling the model with phages, if people put too many in (often children), they would not shut properly."	Delivery team observation	-
